# Aesthetic Refinements in Male Chest Lifting

**DOI:** 10.1093/asjof/ojad021

**Published:** 2023-02-23

**Authors:** Ryan E Austin, John Milkovich, Frank Lista, Jamil Ahmad

## Abstract

The authors describe aesthetic refinements to the approach for male chest lifting in male patients with grade 3 gynecomastia and/or significant chest skin excess. An inferior pedicle is used to transpose the nipple–areolar complex allowing preservation of pigment and sensation, liposuction and direct excision are used to reduce volume and excess skin, and the resulting curvilinear scar along the inferior and lateral border of the chest provide a more masculine appearance. Early experience with this technique has shown it to be safe and effective. Perioperative management and the detailed steps of the procedure are outlined.

**Level of Evidence: 5:**

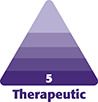

The incidence of gynecomastia—a condition characterized by the benign proliferation of glandular breast tissue in males^1^—is rising, affecting 50% to 60% of adolescents and up to 70% of adults.^2^ In some cases, surgery is required to address this excess tissue improving the aesthetics of the male chest. There has been a 66% increase in the number of total annual surgeries performed from 2020 to 2021 (17,289 and 28,689 procedures, respectively).^3^ Simon et al developed a surgical classification of gynecomastia based on the degree of breast enlargement and the skin redundancy.^4^ We previously described our experience in treating grades 1, 2A, and 2B cases of gynecomastia using power-assisted liposuction and the pull-through technique, which continues to be our approach for these cases.^4,5^ However, grade 3 cases of gynecomastia exhibiting marked breast enlargement and significant chest skin excess present additional anatomical challenges that must be addressed including excess skin, malpositioned nipple–areolar complex (NAC), and poor chest shape, which necessitates the removal of skin from the chest and the repositioning of the NAC.^4^

A variety of surgical techniques have been described to manage cases of grade 3 gynecomastia and/or significant chest skin excess after massive weight loss.^6-16^ Removal of excess skin results in different scar patterns and locations, and the repositioning of the NAC can be done using a pedicle or free nipple graft. Previous surgical techniques to remove excess skin involve a horizontal incision that extends from the central chest to its lateral border.^13^ Another technique employs a boomerang incision coupled with J-torsoplasty to minimize vertical and horizontal skin laxity.^14^ While these techniques afford substantial skin removal from the chest and upper abdomen, the disadvantage is a more conspicuous scar. In these grade 3 cases, free nipple grafting is also commonly used but this increases risks including hypopigmentation and loss of sensation of the NAC.^16^ Alternatively, the use of an inferior pedicle to transpose the NAC reduces these postoperative risks and has been described by Kornstein and Cinelli, in combination with a skin excision pattern resulting in a scar that extends laterally from the inframammary fold to the lateral mammary fold.^12^

Over the past few years, our approach to these treating grade 3 cases of gynecomastia has evolved to use an inferior pedicle to transpose the NAC, and liposuction and direct excision to reduce volume resulting in a curvilinear scar along the inferior and lateral border of the chest providing a more masculine appearance. In addition to contouring the anterior chest, this approach allows for contouring the lateral chest with a resulting scar that is placed in a natural transition zone along the inferolateral border of the pectoralis major muscle. In this study, we review our early experience and outcomes using this approach for grade 3 cases of gynecomastia. The guiding principles outlined in the Declaration of Helsinki were strictly adhered to throughout the study. Written consent was provided, by which the patients agreed to the use and analysis of their data.

## PREOPERATIVE CONSIDERATIONS

Patients should be evaluated to rule out pathological causes of gynecomastia and/or the presence of breast masses. Patients who have grade 3 gynecomastia or those after massive weight loss with significant chest skin excess and inferior malposition of the NAC are ideal candidates for this approach. Patients must be nonsmokers or must have quit smoking for 4 weeks prior to surgery. Prior to surgery, patients should have a stable weight with a body mass index of <35 kg/m^2^.

In the preoperative area, patients are started on our perioperative warming protocol, which is continued intra- and postoperatively.^17-19^ One hour prior to surgery, patients are premedicated to minimize opioid requirements and reduce postoperative nausea and vomiting (Table 1).^18,19^ Compression stockings and sequential compression devices are placed on the lower extremities prior to the induction of anesthesia to reduce the risk of venous thromboembolism (VTE). Chemoprophylaxis for VTE is utilized in very high-risk patients (Caprini/Davison risk assessment model score >7), or if male chest lift is performed in combination with abdominoplasty.^18-20^

**Table 1.** Perioperative Medication Regimen

Medication	Dose and route	Start	Frequency	Duration
Acetaminophen (Johnson & Johnson, Markham, Ontario, Canada)	1000 mg PO	1 h preoperatively	Q 8 h	5 days
Celecoxib (Pfizer, Kirkland, Quebec, Canada)	200 mg PO	1 h preoperatively	Q daily	5 days
Pregabalin (Pfizer, Kirkland, Quebec, Canada)	1st dose: 50 mg PO Subsequent doses: 25 mg PO	1 h preoperatively	TID	5 days
Ondansetron (Novartis Pharmaceuticals Canada Inc., Dorval, Quebec, Canada)	8 mg PO	1 h preoperatively	TID	1 day
Hydromorphone	1-2 mg PO	Postoperatively	Q 6 h PRN pain	7 days
Arnica montana 12C (Boirion, Saint-Bruno-de-Montarville, QC, Canada)	5 pellets PO	Postoperatively	TID	10 days
Dalteparin sodium^a^ (Pfizer, Kirkland, Quebec, Canada)	5000 IU SC	Postoperatively day 1 morning	Q daily	14 days

IU, international unit; PO, by mouth; PRN, as required; Q, every; SC, subcutaneous; TID, three times a day. ^a^Chemoprophylaxis for venous thromboembolism is utilized in very high-risk patients (Caprini/Davison risk assessment model score >7), or if male chest lift is performed in combination with abdominoplasty.

## SURGICAL TECHNIQUE

The operative sequence male chest lift surgery is outlined in Table 2. A detailed demonstration of the procedure can be accessed in the Video.

**Table 2.** Operative Sequence for Aesthetic Male Chest Lift

Step	Details
1	Markings
2	Simultaneous separation and tumescence
3	SAFE Lipo liposuction (if needed)
4	De-epithelialization and dissection of NAC
5	Excision of chest tissue
6	Suprafascial elevation and advancement of superior flap
7	Progressive tension sutures (if needed)
8	Three-point suture to close deep and superficial fascial system of superior and inferior flaps
9	Closure of curvilinear incision with superficial sutures and skin staples
10	Mark new position for NAC
11	De-epithelialize and incise skin at new position of NAC and inset with inverted deep dermal and intradermal sutures
12	Application of dressings and compression garment

NAC, nipple–areolar complex; SAFE, separation, aspiration, and fat equalization.

**Figure 1.**
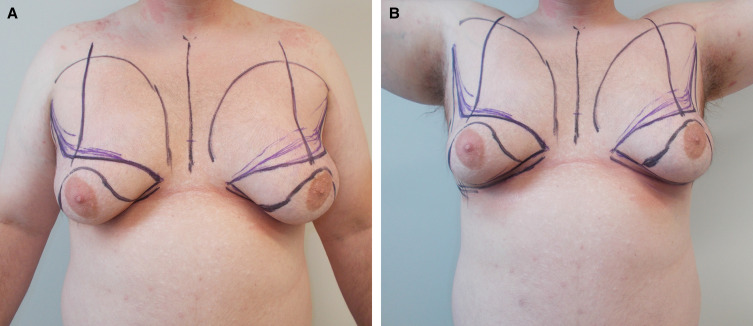
(A, B) Preoperative markings of a 34-year-old male who underwent male chest lift surgery using an inferior pedicle to reposition the nipple–areolar complexes with a curvilinear incision.

**Figure 2.**
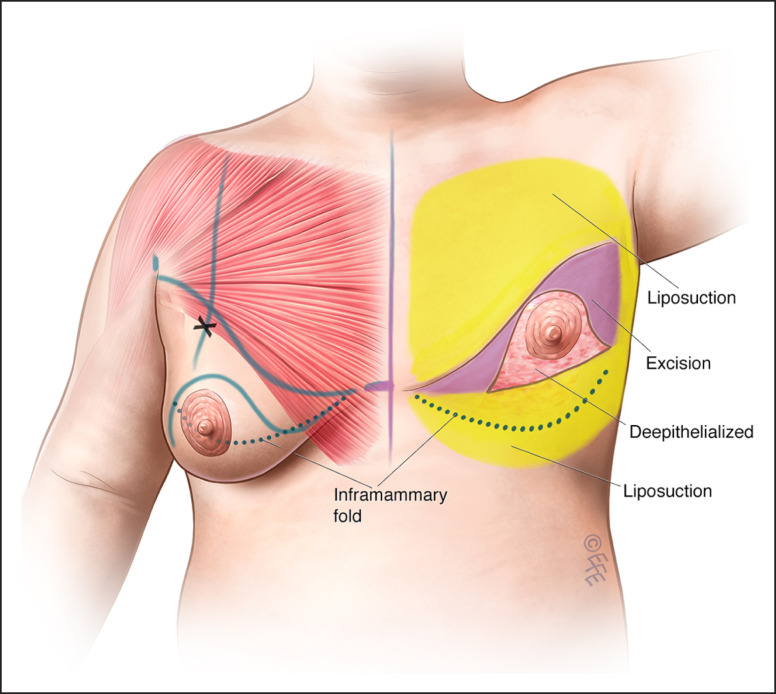
Curvilinear marking pattern. Artwork created by and published with permission from Dr Levent Efe, CMI.

With the patient in the standing position, a curvilinear skin excision pattern is marked, so the resulting curvilinear scar will be along the inferior and lateral border of the chest (Figures 1, 2). The superior extent of the incision should be made with inferior tension and will be the approximate location of the curvilinear scar. The inferolateral extent of the incision should be made of the skin can be closed after the excision; this incision is typically superior to the inframammary fold. In addition, bilateral chest plumb lines and the midline of the chest are marked to help determine the new position of the NACs. The new position of the NACs should be at the height of the inflection point of the inferolateral border of the pectoralis major muscle and this is transposed and marked on the sternum as a reference point and bilateral chest plumb lines passing through this inflection point are marked vertically. The final position of the NACs will be marked intraoperatively. SAFELipo (Separation, Aspiration and Fat Equalization) liposuction is used to reduce chest volume, including the lateral chest.^21^ Specifically, the simultaneous separation and tumescence is performed using a 4-mm basket tip cannula,^22^ followed by power-assisted liposuction (MicroAire Surgical Instruments, Charlottesville, VA) with a 4-mm three hole tip cannula in the planned areas for resection, the upper and lateral chest wall, the upper abdomen, and deep to the pedicle. Following aspiration, fat equalization is performed using a 4-mm basket tip cannula off-suction with an emphasis placed on discontinuously undermining and releasing the zones of adherence of the central and lateral chest and inframammary fold, which facilitates redraping of the chest skin.

Intraoperatively, the inferior pedicle is designed to maintain at least a 1:1 length-to-base ratio. An inferior pedicle is de-epithelialized, and the NAC is transposed to lay at the inferolateral border of the pectoralis major muscle. The superior skin is elevated in the subglandular plane without disrupting the pectoralis fascia, and advanced over the pedicle, while the inferior skin is advanced superiorly and medially. Progressive tension sutures with #1 Vicryl (Ethicon Inc., Somerville, NJ) are performed when there is significant dead space between the flap and the pectoralis fascia, and drains are not used.

The new position of the NACs is marked at the junction of the breast meridian and a line at the level previously marked on the sternum. The skin at the new position of the NACs is excised in a circular pattern with a 20-mm diameter and the NAC is inset. The wounds are closed in layers.

## POSTOPERATIVE CARE

Patients continue a multimodal postoperative oral analgesia protocol.^19^ All patients are seen for routine postoperative follow-up on postoperative day 1, then at day 5 or 6 for removal of skin staples, and they are seen at 2 weeks and 1 month postoperatively. Patients are instructed to wear their postsurgical compression garment for 1 month postoperation^19^ and Lipo foam (ClearPoint Medical, Lachine, QC, Canada) is used underneath this compression garment for the first 2 weeks postoperatively. Patients begin daily chest massage with arnica gel after removal of staples. Once the incision is healed, scar care with silicone sheeting is started and continues for 6 months postoperatively. After 1 month, patients return for routine checks at 3, 6, and 12 months postoperatively.

## EXPERIENCE AND OUTCOMES

**Figure 3.**
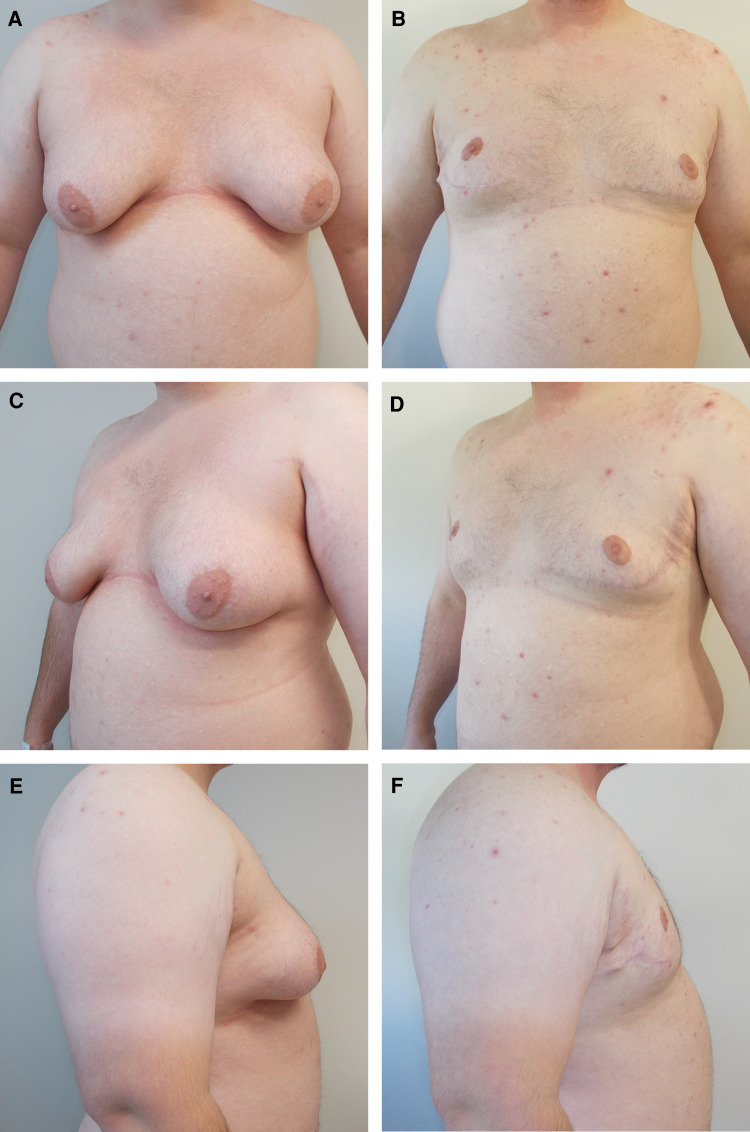
(A, C, E) Preoperative and (B, D, F) 12-month postoperative photographs of a 34-year-old man who underwent male chest lift surgery with a curvilinear incision using an inferior pedicle to reposition the nipple–areolar complexes. He had 232 g excised from the right and 333 g excised from the left and liposuction of the chest with aspiration of 1250 mL from each side. His preoperative markings are shown in Figure 1 and his surgery is shown in the Video.

**Figure 4.**
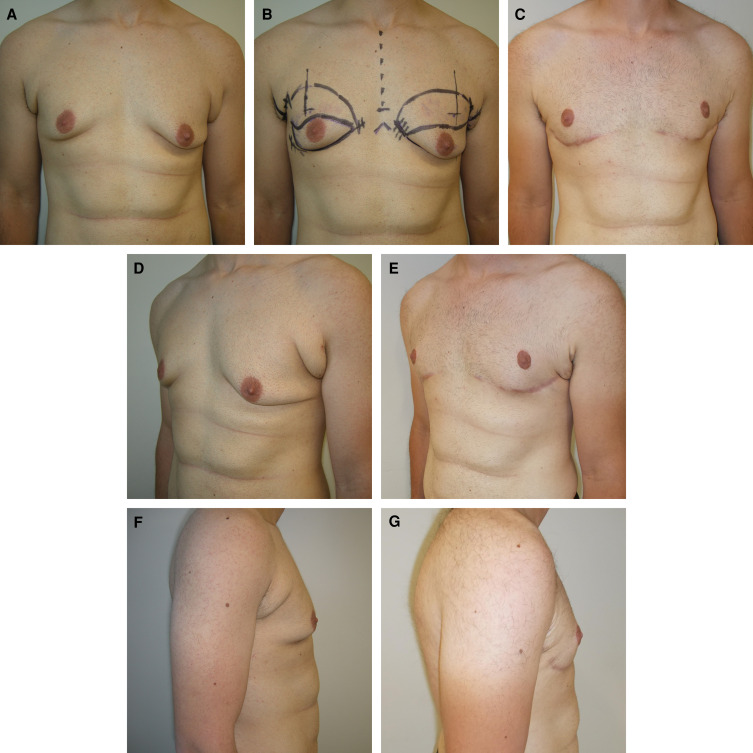
(A, B, D, F) Preoperative and (C, E, G) 13-month postoperative photographs of a 20-year-old male who underwent male chest lift surgery with a curvilinear incision using an inferior pedicle to reposition the nipple–areolar complexes. He had 68 g excised from the right and 98 g excised from the left and liposuction of the chest with aspiration of 100 mL from each side.

We performed a retrospective review of 9 consecutive male patients with grade 3 gynecomastia and/or significant chest skin excess after massive weight loss who underwent surgery from June 2019 to November 2021 (Figures 3, 4). The mean age was 35 years (range 20-55 years) and the mean BMI was 32.1 kg/m^2^ (range, 24.4-40.0 kg/m^2^). The mean operative time was 123 min (range, 91-172 min), the mean total tissue excised per side was 197 g (range, 35-667 g) and the mean total liposuction volume per side was 356 mL (range, 0-1250 mL). Three cases (33.3%) were performed in combination with other body contouring procedures. Eight patients (88.9%) had primary surgery, while 1 patient had secondary surgery (11.1%). The average length of follow-up was 18.7 months (range, 2.1-37.8 months; Table 3).

**Table 3.** Patient Demographics and Procedural Characteristics

Patient demographic/procedural characteristic	Mean (range)/number (percent)
Age (years)	35 (20-55)
Sex	Male: 9 (100.0%) Female: 0 (0.0%)
BMI (kg/m^2^)	32.1 (24.4-40.0)
Tissue weight per side (g)	197 (35-667)
Liposuction volume per side (mL)	356 (0-1250)
Combined procedures	3 (33.3%)
Length of surgery (minutes)	123 (91-172)
Length of follow-up (months)	18.7 (2.1-37.8)
Number of previous chest surgeries	None: 8 (88.9%) 1 surgery: 1 (11.1%)

BMI, body mass index.

One patient had delayed wound healing (11.1%) and 1 patient (11.1%) had scar revision. All patients reported that NAC sensitivity was preserved. The aesthetic outcomes were good, and patients reported being satisfied with the results. While these results are encouraging, the results of this study are limited by its small sample size.

## CONCLUSIONS

Based on our early experience, this novel male chest lifting approach for grade 3 cases of gynecomastia and/or significant skin excess after massive weight loss has been safe and effective. This approach allows preservation of pigment and sensation of the NAC and results in a curvilinear scar that is creates a more masculine appearance of the chest.

## Supplementary Material

ojad021_Supplementary_Data
